# Antiproliferative and pro-apoptotic activity of eugenol-related biphenyls on malignant melanoma cells

**DOI:** 10.1186/1476-4598-6-8

**Published:** 2007-01-18

**Authors:** Marina Pisano, Gabriella Pagnan, Monica Loi, Maria Elena Mura, Maria Giovanna Tilocca, Giuseppe Palmieri, Davide Fabbri, Maria Antonietta Dettori, Giovanna Delogu, Mirco Ponzoni, Carla Rozzo

**Affiliations:** 1 Bio-molecular Chemistry Institute, National Research Council, Sassari, Italy; 2 Differentiation Therapy Unit, Laboratory of Oncology, "G.Gaslini" Children's Hospital, Genova, Italy

## Abstract

**Background:**

Malignant melanoma is one of the most aggressive skin cancer and chemotherapeutic agents currently in use are still unsatisfactory. Prevention and early diagnosis are the only effective tools against this tumour whose incidence and mortality rates are highly increased during the last decades in fair skin populations. Therefore the search for novel therapeutic approaches is warranted. Aim of this work was to identify and test new compounds with antiproliferative and cytotoxic activity on melanoma cells. We tested eugenol together with six natural and synthetic eugenol-related compounds for their capability to inhibit cell growth on primary melanoma cell lines established from patients' tissue samples.

**Results:**

Eugenol and isoeugenol monomers and their respective *O*-methylated forms did not show to inhibit melanoma cells proliferation. Conversely, the dimeric forms (biphenyls) showed some antiproliferative activity which was mild for dehydrodieugenol, higher for its *O,O'-*methylated form (*O,O'*-dimethyl-dehydrodieugenol), and markedly pronounced for the racemic mixture of the brominated biphenyl (*6,6'*-dibromo-dehydrodieugenol) (S7), being its enantiomeric form (*S*) the most effective compared to the other compounds. Such activity resulted to be selective against tumour cells, without affecting cultured normal human skin fibroblasts. Dose and time dependence curves have been obtained for the enantiomeric form S7-(*S*). Then IC_50 _and minimal effective doses and times have been established for the melanoma cell lines tested. TUNEL and phosphatidylserine exposure assays demonstrated the occurrence of apoptotic events associated with the antiproliferative activity of S7-(*S*). Cytotoxic activity and apoptosis induced by treating melanoma cells with eugenol-related biphenyls was partially dependent by caspase activation.

**Conclusion:**

Our findings demonstrate that the eugenol related biphenyl (*S*)-*6,6'*-dibromo-dehydrodieugenol elicits specific antiproliferative activity on neuroectodermal tumour cells partially triggering apoptosis and its activity should be further investigated on *in vivo *melanoma models in order to evaluate the real anticancer effectiveness on such tumour.

## Background

Malignant melanoma arises from the malignant transformation of epidermal melanocytes due to both environmental and genetic factors [1,2]. Melanoma is the most rapidly increasing malignancy in the white population [3] and, at the present, prevention and early detection represent the only effective approach to reduce its incidence. Little progress has been made in medical treatment of melanoma because of the absence of effective systemic therapies. Chemotherapy is not giving significant benefits and it is often associated to severe toxicity [4], while immunotherapy and vaccines are promising but still ineffective [5]. Patients with metastatic melanoma present a median survival of 4–10 months, depending on the involvement of the anatomic site (patients with disease confined to skin or lymph nodes have an overall survival longer than those with visceral involvement) [6].

It is therefore of primary interest to search for new therapeutic agents able to contrast this aggressive tumour. One of the developing strategies is to test some naturally occurring compounds whose chemopreventive properties in cancer therapy are already known [7].

Eugenol is a natural phenolic compound that is the main component of clove oil and it is present in reasonable amounts in several other spices like basil, cinnamon and bay leaves. It is used as antiseptic, analgesic and anti-bacterial agent in traditional medicine in Asia as well as in dentistry as main ingredient of cavity filling cement. Several biological activities of eugenol have been described in literature [8,9] and it has been proved not to be carcinogenic neither mutagenic [10,11]. It possesses antiviral activity *in vitro *and *in vivo *against human herpesvirus [12]. Recently, eugenol was found to induce apoptosis in mast cells [13], melanoma cells [14] and HL-60 leukemia cells [15]. Moreover, it has been reported that dimers of eugenol related compounds have a better antioxidant activity than the original monomers [16-18] and that bis-eugenol shows stronger apoptotic activity than eugenol on leukemic cells [15]. Dimers of eugenol related compounds are hydroxylated biphenyls such as magnolol and honokiol, being the latters the main constituents of the stem bark of *Magnolia officinalis*, whose biological properties are well known in literature. Indeed, they both have antioxidant and antidepressant properties [19,20] and recent reports have demonstrated that honokiol have antiangiogenic and antitumoural activity inducing apoptosis [21,22].

On this basis, we screened a panel of eugenol related compounds in order to verify their possible ability to interfere with melanoma cells growth, as a first step toward the development of new therapeutic drug. We decided to compare eugenol and six related compounds differing for structure (monomeric or dimeric), chemical groups (hydroxylated, *O*-methylated and/or brominated) and specific conformational structure [(*S*) or (*R*) enantiomeric forms] for antitumour capability. We demonstrated that the byphenyl structure, the presence of bromine substitution and a specific stereo configuration of the molecule are important for the antiproliferative activity and induction of apoptotic cell death on melanoma cell lines.

## Results

### Effect of eugenol derivatives (S1–S7) on the proliferation of melanoma cell lines

**Figure 1:**
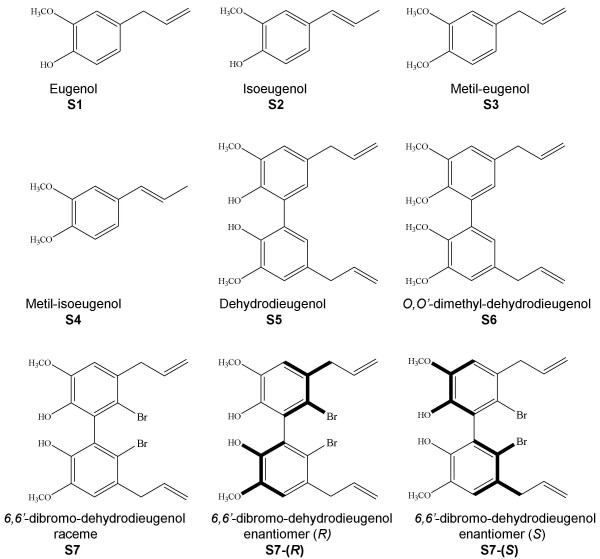
Chemical structures of eugenol and related compounds.

**Figure 2:**
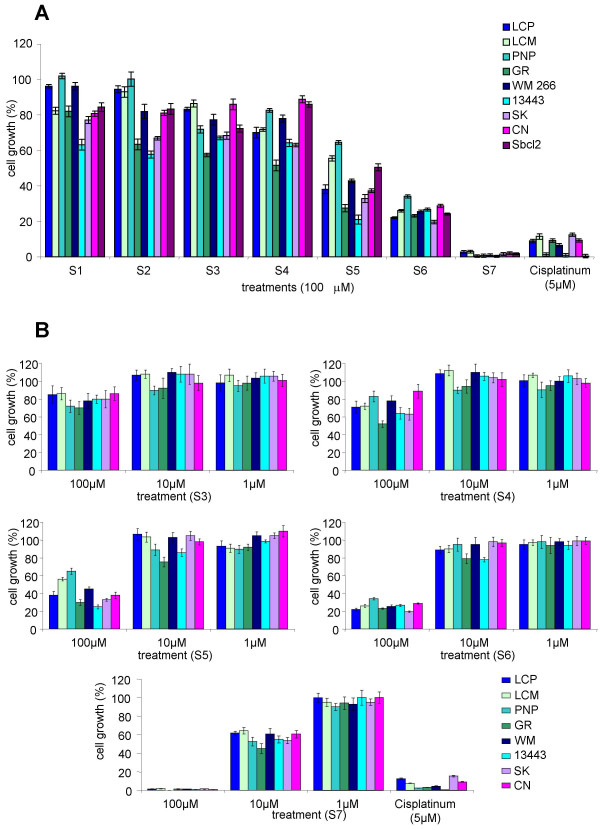
**Effect of eugenol derivatives on the growth of human melanoma cell lines**. Nine melanoma cell lines were cultured (**A**) in presence of 100 μM or (**B**) with various concentrations of each compound (S1–S7), for 6 days and cell proliferation was estimated as described in Material and Methods. Results are expressed as percent of cell growth and represent the average (± standard deviation) of triplicate cultures performed twice. 5 μM Cisplatinum was used as cytotoxic agent positive control.

Cell proliferation assays were performed to test the possible cytotoxicity of eugenol related compounds. As a first screening, nine melanoma cell lines (see Methods section for cell lines details) were grown up to 6 days in the presence of 100 μM of each of the seven compounds S1 to S7, listed in Figure 1. None of the nine melanoma cell lines was inhibited by the treatment of 100 μM eugenol (S1) or isoeugenol (S2) (Figure 2). As shown in Figure 2A, methyl-eugenol (S3) and methyl-isoeugenol (S4) treatments slightly inhibited melanoma cells growth (of about 20–40%). Dimeric forms were more effective. In particular, dehydrodieugenol (S5) decreased cell growth rate of about 40–60%, *O,O'*-dimethyl-dehydrodieugenol (S6) caused a consistent growth inhibition of about 70–80%, whereas the *6,6'*-dibromo-dehydrodieugenol (S7) induced a quite complete growth inhibition (about 100%) in all the nine melanoma cell lines (Figure 2A). Treatment with the latter compound showed an efficacy on cell proliferation better than the administration of 5 μM cisplatinum, a chemotherapeutic drug known as potent cytotoxic agent on cancer cell lines. On the basis of these results we decided to further investigate on the antiproliferative activity of compounds S3 to S7.

#### Dose dependence of S3–S7

Proliferation assays were then performed using three different concentrations of the eugenol-related compounds showing cytotoxic activity. Formerly, melanoma cell lines were treated with 100, 10, and 1 μM of each S3–S7 and proliferation rates were evaluated after 6 days as described in the Materials and Methods section (Figure 2B). Cell growth inhibition at 100 μM of these compounds somehow reproduced the data of Figure 2A. At lower concentration (10 μM) S3, S4, S5, and S6 showed only partially specific inhibition of melanoma cell growth, while S7 confirmed its activity on all the tested cell lines (most of them presented a 80% or lower of growth rate, with few, more sensitive, cell lines, such as GR and 13443, whose growth rate was around the 50%). None of the compounds tested at 1 μM final concentration showed any significant activity. These results suggested that compounds S3–S6 have a much weaker anti-proliferative effect to be further tested in *in vivo *assays. Conversely, our interest has been focused on the potential biological activity of the S7 dimer. The S7 compound is a chiral molecule (Figure 1) therefore two atropo-enantiomers are evidenced at room temperature. Often, the two stereoisomers can show different biological activity, for this reason we decided to test the activity of both the enantiomers S7-(*S*) and S7-(*R*) on tumour cells.

#### Enantiopure form S7-(*S*) is more active than both S7-(*R*) and their racemic mixture

**Figure 3:**
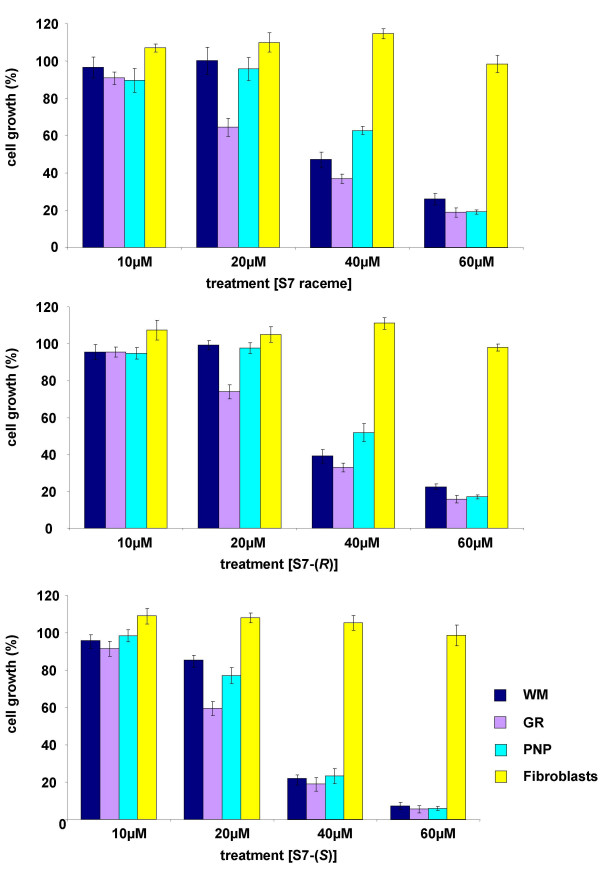
**Dose-dependent S7 antiproliferative activity: comparison of raceme mixture with the enantiopure forms S7-(*R*) and S7-(*S*)**. Three melanoma cell lines and one short term culture of human fibroblasts were treated with various concentrations of S7, S7-(*R*) and S7-(*S*) for 3 days. Cell proliferation was estimated as described in Material and Methods. Results are expressed as percentage of cell growth and represent the average (± standard deviation) of triplicate cultures performed twice.

The dose-dependent effects on three melanoma cell lines (WM, GR, PNP) and on normal fibroblasts short term culture (as control) were tested using S7 racemic mixture, S7-(*R*) and S7-(*S*). Graphics reported in Figure 3 show that the S7-(*S*) enantiomeric form was more active on inhibiting melanoma cell proliferation than the two other forms and that normal fibroblasts were not affected by these treatments up to the concentration of 60 μM. IC_50 _values for each form of S7 on the melanoma cell lines tested are reported in Table 1.

**Table 1:** IC_50 _for S7, S7-(*R*) and S7-(*S*)

**IC_50 _[μM]**			
Cell lines	S7 raceme	S7-(*R*)	S7-(*S*)

WM (MM)	38,5	36	27
GR (MM)	30,0	31,5	23
PNP (MM)	46,0	41	29
GILIN (NB)	n.d.	n.d.	19
LAN-5 (NB)	n.d.	n.d.	16

Legend: MM (Malignant Melanoma)

NB (Neuroblastoma)

n.d. (not done)

To validate the efficacy of S7-(*S*) as novel putative anticancer agent for neuroectoderma-derived tumours, we tested its anti-proliferative activity also on neuroblastoma (NB) cells, the pediatric solid tumour sharing with melanoma the origin from neural crest cells. As reported in Table 1, S7-(*S*) was slightly more efficient on NB cells showing IC_50 _values less than 20 μM after 5 days of treatments.

### Time dependence and anticancer specificity of S7-(*S*)

**Figure 4:**
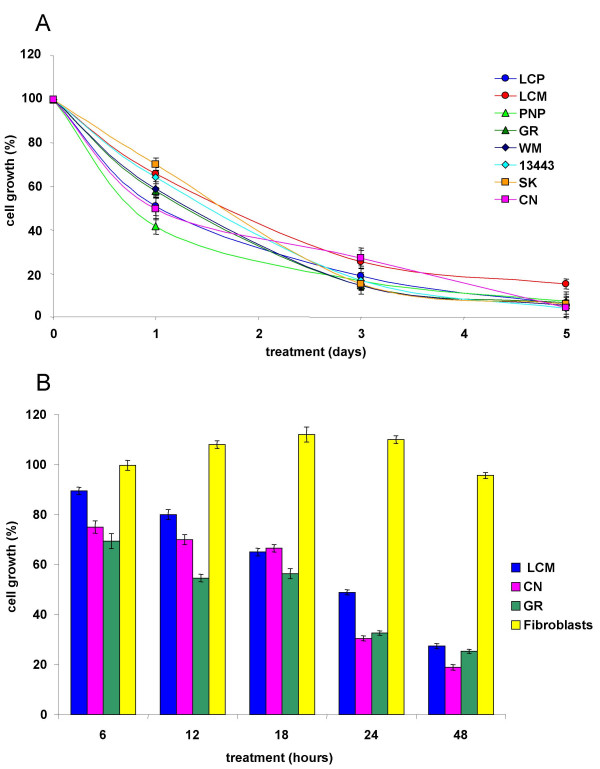
**Time-dependent antiproliferative effect of S7-(*S*) on melanoma cells**. **(A) **Eight melanoma cell lines were treated with 50 μM S7-(*S*) and cell proliferation was estimated at the different points, as described in Material and Methods. Results are expressed as percentage of cell growth and represent the average (± standard deviation) of triplicate cultures performed twice. **(B) **S7-(*S*) wash-out assay. Three melanoma cell lines and one short term culture of human fibroblasts were treated with 50 μM S7-(*S*) for various times (6–12–18–24–48 hours), then washed and incubated with S7-(*S*) free medium. Cell proliferation was estimated 48 hours after initiation of treatment, as described in Material and Methods. Results are expressed as percentage of cell growth and represent the average (± standard deviation) of triplicate cultures performed twice.

Time-course experiments using 50 μM S7-(*S*) up to 5 days showed that its anti-proliferative activity was already evident after 24 hours of treatment (30–60% of growth inhibition), reaching 90–100% of growth inhibition after 3 treatments in the 5^th ^day (Figure 4A).

To simulate the activity of S7-(*S*) *in vivo*, we tested the response of melanoma cells to the drug after very short exposures. Indeed, wash-out experiments using 50 μM S7-(*S*) up to 48 hours better defined the action time window of such compound establishing that a single administration of S7-(*S*) is sufficient to inhibit melanoma cell growth up to 80% for 48 hours with no effect on cell growth of normal fibroblasts (Figure 4B).

**Figure 5:**
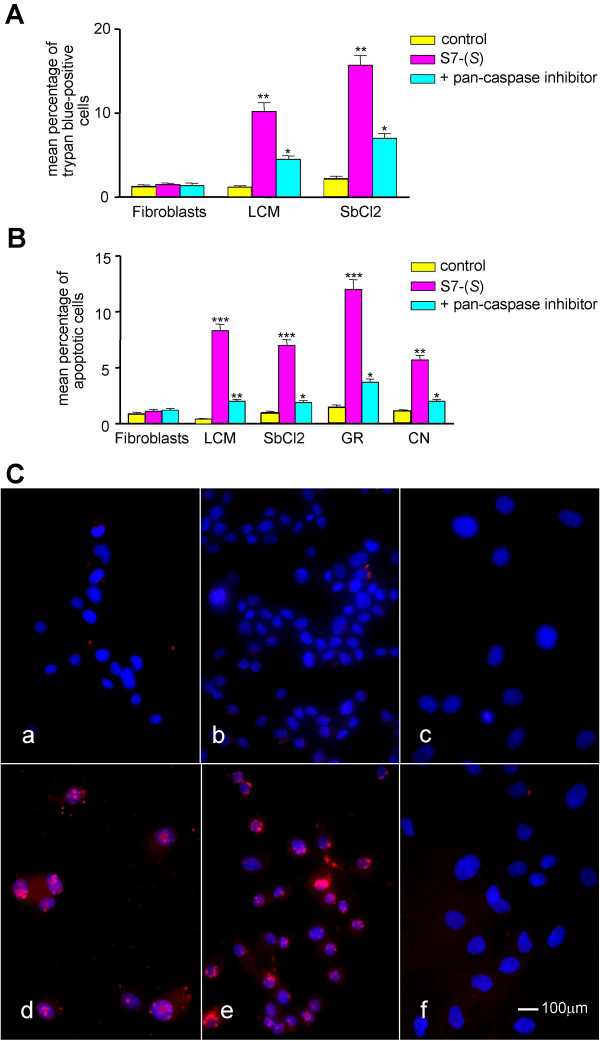
**Effect of S7-(*S*) on cell viability and apoptosis in melanoma cells**. **(A) **Melanoma cells and normal fibroblasts were incubated with 50 μM S7-(*S*) or solvent (control) for 24 hours, and cell death was determined by trypan blue staining. Results are expressed as mean number of trypan blue-positive cells in triplicate cultures from two independent experiments. Cells were also pre-treated for 1 hour with the pan-caspase inhibitor before S7-(*S*) treatment. *P *values for S7-(*S*) versus control and for inhibitor versus S7-(*S*) were calculated by Student's t-test with Welch correction: *, *P *< .05; **, *P *< .01. **(B) **Effect of S7-(*S*) on phosphatidylserine exposure on melanoma cells. Human fibroblasts and melanoma cell lines were incubated in the presence or absence of 50 μM S7-(*S*) for 24 hours. Cells were also pre-treated for 1 hour with the pan-caspase inhibitor before S7-(*S*) treatment. Cells were double stained with annexin V-FITC to detect phosphatidylserine exposure and propidium iodide to detect DNA and analysed by flow cytometry. Results are expressed as mean percentage of apoptotic cells from three independent experiments. *P *values for S7-(*S*) versus control and for inhibitor versus S7-(*S*) were calculated by Student's t-test with Welch correction: *, *P *< .05; **, *P *< .01; ***, *P *< .001. **(C) **Effect of S7-(*S*) on DNA fragmentation in melanoma cells. GR-mel (a, d), Sbcl2 (b, e) melanoma cell lines and human fibroblasts (c, f) were treated with 50 μM (d, e, f) S7-(*S*) or grown in S7-(*S*) free medium (control a, b, c) for 24 hours and apoptotic cells (red) were stained by the TUNEL assay. Cell nuclei (blue) were stained with DAPI.

We then examined the effect of S7-(*S*) on the viability of cultured melanoma cells by trypan blue staining. Melanoma cells and fibroblasts were incubated with 50 μM S7-(*S*) for 24 hours and their viability was assessed. The results showed a statistically significant cytotoxic activity of S7-(*S*) on melanoma cells without affecting non-transformed cells (Figure 5A). Similar results were obtained with the other melanoma and NB cell lines (data not shown).

### Effects of S7-(*S*) on apoptosis in melanoma cell lines

To determine whether the observed S7-(*S*)-induced reduction in viability of melanoma cells occurred via induction of apoptosis, we used TUNEL staining to measure DNA fragmentation and phosphatidylserine exposure as an early hallmark of apoptotic cell death in treated cells. As evidenced in Panels *d *and *e *of Figure 5C, a small, but consistent proportion of TUNEL-positive melanoma cells (15–20% apoptotic fraction) was detected after 24 hours of treatment with 50 μM S7-(*S*), while no evidences of apoptosis were observed in human fibroblasts (Fig. 5C, panel *f*). Moreover, the same treatment of melanoma cells with S7-(*S*) induced a significant increase in the number of cells expressing phosphatidylserine on cell surface (Figure 5B).

Besides nuclear condensation and appearance of oligonucleosomal DNA, induction of apoptosis may involve activation of aspartate specific cysteine proteases or caspases [23]. Melanoma cells were treated for 24 hours with 50 μM S7-(*S*) in the presence or absence of pan-caspase inhibitor (z-VAD-fmk, 100 μM). Pan-caspase inhibitor significantly reduced the number of trypan blue-positive cells (Figure 5A) and significantly blocked S7-(*S*)-triggered apoptosis (Figure 5B).

## Discussion

To verify the possible anti-proliferative effect of some eugenol derivatives as a first step toward the development of novel putative anticancer agents, we tested eugenol and isoeugenol as well as five eugenol-related compounds for their capability to inhibit cell growth and viability on a panel of melanoma cell lines.

Previous report showed that eugenol, but not its isomer isoeugenol, inhibit melanoma cell proliferation by arresting cells in the S phase of cell cycle and inducing apoptosis [14]. Moreover, eugenol and creosol dimers are widely reported as cytotoxic and antioxidant agents inducing apoptosis on HL-60 leukemia cells [15,17], with much stronger effects than their original monomers. We have previously described the synthesis and purification of the two enantiopure forms of 6,6'-dibromo-dehydrodieugenol and its antinociceptic activity [24,25]. In the present study, we demonstrate that this compound, a brominated dimer of eugenol, is the most potent eugenol-derivative able to show antiproliferative action on melanoma cells, partially triggering apoptosis, and it could be considered a potential candidate to be tested in *in vivo *models as anti-cancer agent. Moreover, we show that the enantiopure form (a*S*) of such compound is much more active, as antiproliferative agent, than both the racemic mixture and the enantiopure (*R*) form. This is a frequent property of chiral compounds whose biological activity is often related to a defined absolute configuration. Stereo-isomeric forms of same compound may show very different properties and bio-activity, like different degree of absorption and clearance, different metabolic pathways and toxicological characteristics [26]. To date, brominated biphenyls have been described to have a role in antimicrobial and antiviral therapies [27,28]. Our findings point out the anti-proliferative properties of eugenol related compounds, whose dimeric forms, conversely from the eugenol monomeric form (which did not show any activity on inhibiting melanoma cell growth), are able to block tumour cell growth. Indeed, none of the nine melanoma cell lines (including Sbcl2 cell line used by Ghosh and collaborators [14]) was inhibited by treatment with 100 μM eugenol (S1) and isoeugenol (S2) (see Figure 2). This contrasting data could be due to the different culture conditions used in the laboratories. In our experiments, the anti-tumour capability notably increased in the methylated and brominated dimeric forms of eugenol, which indeed show better cytotoxic activity than the monomeric forms. The importance of the dimeric structure is also shown by other compounds of natural origin, such as the hydroxylated biphenyls magnolol and honokiol, whose biological properties are well known in literature (honokiol has been proven to be a potent anti-angiogenic and anti-tumoural compound inducing apoptosis [21,22]).

Moreover, the S7-(*S*) compound seems to have a quite specific activity on tumour cells, since it is not toxic to normal human diploid fibroblasts when used at the same conditions that completely arrest melanoma cells growth and induced cell death. Wash-out experiments were performed to reproduce *in vivo *like conditions, treating cells with a single dose of the compound to be tested, and washing it out after increasing time intervals, in order to mimic drug processing and clearance occurring in tumour tissue [29]. Such experiments demonstrated that a single dose of 50 μM S7-(*S*) drastically inhibits melanoma cell growth up to 80% after 48 hours of treatment without affecting cell growth in normal cells (see Figure 4B).

We finally addressed the question whether inhibition of cell proliferation and induction of cell death in melanoma cells by S7-(*S*) is mediated by apoptosis. Although, our findings strongly suggest a pivotal role of caspases in S7-(*S*)-triggered apoptosis, further studies will better clarify the role of upstream signals – between drug exposure and caspase activation – or the involvement of cell death mechanisms other than apoptosis, such as autophagy and mitotic catastrophe [30,31].

## Conclusion

In conclusion this work demonstrates that the biphenyl eugenol-derivative enantiomer (*S*)-*6,6'*-dibromo-dehydrodieugenol, is an effective cytotoxic agent for melanoma and neuroblastoma cells and it is endowed with apoptotic inducing capability. Moreover its action is selective on tumour cells not interfering with normal cells growth. Our data support the effectiveness of biphenyl as dimeric structures with specific substitute and stereo configuration in inhibiting the malignant proliferation of neuroectodermal tumour cells *in vitro*. Further investigation of S7-(*S*) in mouse cancer models will contribute to better understand its *in vivo *activity against malignant cells and its real toxicity in normal tissues. Assessment of the molecular mechanism underlying the activity of this compound as well as biological test of similar substances to be active at lower concentration will represent the aims of future research efforts.

## Methods

### Cell lines

Malignant melanoma cell lines WM266-4 (WM), SK-Mel-28 (SK), LCP-Mel (LCP), LCM-Mel (LCM), PNP-Mel (PNP), CN-MelA (CN), 13443, GR-Mel (GR) were kindly provided by Drs. D. Castiglia and S. D'Atri at the Institute "Dermopatico dell'Immacolata" in Rome. They were established as primary short term cell cultures starting from tumour samples of donors patients with documented diagnosis of malignant melanoma after obtaining their informed consent, as previously described [32]. SbCl2 melanoma cell line [33] and the human Neuroblastoma (NB) cell lines, GI-LI-N [34] and LAN-5 [35], were used between passages 50 and 75.

Short term cultures of normal human fibroblasts from healthy donors were established at National Cancer Institute in Naples and at Gaslini Children's hospital and used as controls.

Cells were cultured to confluence in tissue culture flasks using either Dulbecco's minimal essential medium (DMEM) or RPMI medium (Invitrogen, Carlsbad, CA) supplemented with 10% FBS and penicillin/streptomycin [100 IU (50 μg)/ml] in a humidified 5% CO_2 _atmosphere at 37°C, as previously described [36,37,33].

### Eugenol and derivatives

Eugenol (S1) and isoeugenol (S2) were purchased from Sigma-Aldrich (St. Louis, MO), purity 99% and 98% respectively. Isoeugenol was a 90:10 mixture of trans:cis isomeric forms. Eugenol and isoeugenol derivatives S3–S7 (Fig. 1) were synthesized at the C.N.R., Bio-molecular Chemistry Institute in Sassari, as previously described [24]. S1–S4 were solved in ethanol 99% while S5–S7 in DMSO, all at a stock dilution of 100 mM, and then used for cells treatments at concentration between 100 μM and 1 nM in complete medium. Chemical structures and nomenclature of the compounds used are reported in Figure 1.

The chemotherapeutic agents Cisplatinum and Etoposide (Teva Pharma B.V., Holland) were used as cytotoxic positive controls at concentration of 5 μM and 25 μM, respectively, in complete medium.

### Cell proliferation assay

Cells were plated (3 × 10^3^/well) in 96-well plates in their complete medium. After 24 hours, medium was removed and replaced on days 1, 3 and 5 in quadruplicate by the same medium (control) or supplemented with various S1–S7 doses as described in legend of figures. Cells were observed with inverted microscope every 24 hours to check on morphological changes, suffering or cell death. The percentage of cell proliferation was estimated on day 6 by the colorimetric assay of Kueng et al. [38] modified as follows: cells were fixed for 20 min at room temperature with 4% paraformaldehyde (PFA), stained with 0.1% crystal violet in 20% methanol for 20 min, washed with PBS, solubilized with 10% acetic acid and read at 595 nm in a microplate reader (SpectraFluor Plus, Tecan, Austria).

To assess the impact of drug exposure time on cell proliferation, washout experiments were also performed according to the method shown by Keshelava et al. [39]. Briefly, S7-(*S*) containing medium was removed from the cultures after 6–12–18–24 hours by two PBS washings. Cells were subsequently covered with fresh drug-free medium and incubated until 48 hours and cell proliferation rate was evaluated by the same colorimetric assay.

### Cell viability assay

Following the indicated treatments (see legend of Figure 5), cells were incubated with trypan blue, for one minute at 37°C. Trypan blue-positive and total cells were counted per microscope field, for a total of four fields for each combination. The proportion of dead cells was calculated by dividing the number of dead cells by that of total cells/field. Alternatively, viable cell number was counted. In some experiments, cells were cultured for 1 hour with 100 μM Z-VAD-FMK (pan-caspases inhibitor, Sigma Aldrich, St. Louis, MO) before S7-(*S*) treatments, and then viability was evaluated.

### Apoptosis assays

#### TUNEL assay

Melanoma cells (5 × 10^4^/well) were plated in 8 well-chamber slides and, after 24 hours of culture, were treated with S7-(*S*) (50 μM and 100 μM) or 25 μM Etoposide. After a further 24 hours, DNA cleavage was assessed by enzymatic end-labeling of DNA strand breaks using a commercial kit (In Situ Cell Death Detection Kit, Roche, Penzberg, Germany), according to the manufacturer's instructions. Briefly, slides with adherent cells were air-dried and fixed in 4% PFA for 1 hour at room temperature, washed in PBS and permeabilized with 0.1% Triton X-100 and 0.1% sodium citrate for 2 minutes at 4°C; after rinsing, slides were incubated with 50 μl of TUNEL reaction mixture, containing terminal deoxynucleotidyl transferase (TdT) and TMR red-labeled dUTP, in a humidified atmosphere for 1 hour at 37°C in the dark. Rinsed slides were then dyed with 0.2 μg/ml 4',6'-diamidino-2-phenylindole (DAPI) (Vector Laboratories, Burlingame, CA) in PBS, for nuclear counterstaining, and then washed again with PBS. Finally slides were coverslipped with Mowiol 4–88 mounting medium (Calbiochem, San Diego, CA). Cells were analyzed under a Olimpus BX 50 fluorescent microscope with appropriate DAPI and TMR red filter sets. The images were acquired with a high-sensitivity monochrome charge-coupled device (CCD) camera.

#### Phosphatidylserine detection

Phosphatidylserine (PS) exposure was assessed with a human Annexin V- fluorescein isothiocyanate (FITC) Kit (Bender MedSystems, Vienna, Austria). Briefly, cultured and S7-(*S*) treated primary melanoma cells were collected, washed and incubated for 10 minutes with 5 μl Annexin V-FITC, washed once with PBS, resuspended in 190 μl prediluted binding buffer containing 10 μl of a 20 μg/ml propidium iodidide (PI) I stock solution and examined by two colour flow cytometry using a FACScan (Beckton Dickinson, Franklin Lakes, NJ). FITC and PI fluorescence intensities were recorded through a 520–530 (FL1-H) and 575 (FL2-H) nm filters, respectively. At least 10,000 events were collected in each dot plot and analyzed using Cell Quest software (Beckton Dickinson, Franklin Lakes, NJ). Cells that did not stain for either Annexin V-FITC or PI were viable and did not undergo measurable apoptosis; those that stained for Annexin FITC, but not for PI, were in the early stages of apoptosis; cells that stained positive for both Annexin V-FITC and PI were either in the late stages of apoptosis or necrotic, as previously described [40].

### Statistical methods

Results are expressed as mean ± standard deviation. All *in vitro *data are from at least three independent experiments. The statistical significance of differential findings between experimental groups and controls was determined by Student's t-test with Welch correction. These findings were considered significant if two-tailed *P *values were < 0.05.

## Competing interests

The author(s) declare that they have no competing interests.

## Authors' contributions

M Pisano carried out cell cultures, cell proliferation assays, TUNEL assays and drafted the manuscript. G Pagnan carried out cell viability assays and phospatidylserine detection. ML, MEM and MGT participated to cell cultures and cell proliferation assays. G Palmieri contributed to the final drafting and critical revision of the manuscript. DF, MAD and GD performed the chemical synthesis of eugenol-and isoeugenol-derivatives and the resolution of S7. M Ponzoni performed statistical analysis and together with CR conceived of the study, participated in its design and coordination and carried out the final drafting of the manuscript.
